# Repeatability and sensitivity of $T_{2}^{*}$ measurements in patients with head and neck squamous cell carcinoma at 3T

**DOI:** 10.1002/jmri.25134

**Published:** 2016-01-22

**Authors:** Rafal Panek, Liam Welsh, Alex Dunlop, Kee H. Wong, Angela M. Riddell, Dow‐Mu Koh, Maria A. Schmidt, Simon Doran, Dualta Mcquaid, Georgina Hopkinson, Cheryl Richardson, Christopher M. Nutting, Shreerang A. Bhide, Kevin J. Harrington, Simon P. Robinson, Kate L. Newbold, Martin O. Leach

**Affiliations:** ^1^ CR‐UK Cancer Imaging CentreLondonUK; ^2^ Institute of Cancer ResearchLondonUK; ^3^ Royal Marsden NHS TrustLondonUK

**Keywords:** BOLD, MRI, hypoxia, oxygen, head and neck

## Abstract

**Purpose:**

To determine whether quantitation of $T_{2}^{*}$ is sufficiently repeatable and sensitive to detect clinically relevant oxygenation levels in head and neck squamous cell carcinoma (HNSCC) at 3T.

**Materials and Methods:**

Ten patients with newly diagnosed locally advanced HNSCC underwent two magnetic resonance imaging (MRI) scans between 24 and 168 hours apart prior to chemoradiotherapy treatment. A multiple gradient echo sequence was used to calculate $T_{2}^{*}$ maps. A quadratic function was used to model the blood transverse relaxation rate as a function of blood oxygenation. A set of published coefficients measured at 3T were incorporated to account for tissue hematocrit levels and used to plot the dependence of fractional blood oxygenation (Y) on $T_{2}^{*}$ values, together with the corresponding repeatability range. Repeatability of $T_{2}^{*}$ using Bland–Altman analysis, and calculation of limits of agreement (LoA), was used to assess the sensitivity, defined as the minimum difference in fractional blood oxygenation that can be confidently detected.

**Results:**

$T_{2}^{*}$ LoA for 22 outlined tumor volumes were 13%. The $T_{2}^{*}$ dependence of fractional blood oxygenation increases monotonically, resulting in increasing sensitivity of the method with increasing blood oxygenation. For fractional blood oxygenation values above 0.11, changes in $T_{2}^{*}$ were sufficient to detect differences in blood oxygenation greater than 10% (Δ $T_{2}^{*}$ > LoA for ΔY > 0.1).

**Conclusion:**

Quantitation of $T_{2}^{*}$ at 3T can detect clinically relevant changes in tumor oxygenation within a wide range of blood volumes and oxygen tensions, including levels reported in HNSCC. J. Magn. Reson. Imaging 2016;44:72–80.

Tissue oxygenation is an important parameter of the tumor microenvironment that influences both proliferation and angiogenesis.^1^, ^2^ The presence of hypoxic regions within tumors is considered an important cause of treatment failure affecting both radiotherapy and chemotherapy, and adversely affects the prognosis of head and neck squamous cell carcinoma (HNSCC).^3^, ^4^, ^5^ Noninvasive methods to rapidly quantify the spatial distribution and extent of hypoxia within an individual tumor are thus highly desirable in clinical practice to allow modification of treatment strategies in this poorer prognosis group.

Magnetic resonance imaging (MRI) measurements of the transverse relaxation time ( $T_{2}^{*}$) have been proposed as imaging biomarkers of tissue oxygenation status in both preclinical and clinical settings.^3^, ^4^, ^6^, ^7^ Paramagnetic deoxyhemoglobin increases the apparent MRI transverse relaxation rate $R_{2}^{*}$ (=1/ $T_{2}^{*}$) of water in blood and surrounding tissues, which provides the opportunity to image tissue oxygenation at high spatial resolution. The $T_{2}^{*}$ of the vascular space is dependent on fractional blood oxygenation (Y), and can be described by a quadratic function of hematocrit levels (Hct) and magnetic field strength (B_0_).^8^, ^9^ Changes in $R_{2}^{*}$ are used to study brain activity associated with modulated regional brain perfusion (the blood oxygen level dependent or BOLD effect).^10^ However, the strength of the correlation between tissue $R_{2}^{*}$ and tissue oxygen partial pressure (pO_2_), measured using oxygen electrodes, and immunohistochemical detection of the hypoxia marker pimonidazole, has been reported to be only weak to moderate.^11^, ^12^, ^13^ Consequently, quantitative measurements of tissue oxygenation using BOLD have yet to be established.^14^

BOLD measurements can also be performed in combination with hyperoxic gas breathing, whereby the oxy/deoxygenated hemoglobin ratio is altered. The magnitude of changes in BOLD measurements within tumors, on breathing hyperoxic gas relative to air, have been shown to relate to the tumor hypoxic fraction as determined by pimonidazole labelling.^15^ An increase in $T_{2}^{*}$ was found to correlate with inhalation of higher percentages of oxygen in preclinical studies, but the magnitude of signal changes was not proportional to the absolute measured tissue oxygenation.^16^ Preclinical and clinical studies in prostate, cervix, and head and neck cancers have consistently reported increases in tumor tissue $T_{2}^{*}$ in response to hyperoxic gas challenge.^17^, ^18^, ^19^, ^20^

In contrast, studies in breast cancer have shown differing results, as tumors were found to exhibit $T_{2}^{*}$ decreases following hyperoxic challenge but with relatively large magnitude variation in the magnitude of $T_{2}^{*}$ changes.^21^ This discrepancy is thought to relate to differences in tumor biology across histologies.^15^, ^21^, ^22^ Such discordant results may be explained by $T_{2}^{*}$‐weighted signal dependence on physiological factors, including the hematocrit, blood volume (BV), vessel caliber,^23^, ^24^ and the intermittent tumor vessel blood flow.^16^, ^25^ Therefore, the relationship of baseline tumor tissue $T_{2}^{*}$ to tumor hypoxia varies according to the nature of the tumor vasculature and its host hematological status. It therefore should not be surprising that tumor $T_{2}^{*}$ and its response to various tumor challenges (treatment, hyperoxia) varies according to tumor type.

In addition to the tumor vascular microenvironment, quantitation of $T_{2}^{*}$ is also dependent on physiochemical and methodological parameters.^14^, ^26^, ^27^ Macroscopic magnetic field homogeneity resulting from the iterative shimming process affects the repeatability of $T_{2}^{*}$ measurements. The measured $T_{2}^{*}$ value within a voxel is a composite of spin relaxation rates within the intra‐ and extravascular tissue spaces, a consequence of the scanning spatial resolution. Spin relaxation in the extravascular space has a much weaker and variable dependency on blood oxygenation than that for the intravascular space within a tumor.^10^, ^26^

Hypoxia is a common and well‐recognized cause of radioresistance in HNSCC, and therefore MRI‐based measurements of oxygenation in this region are of significant interest.^3^, ^4^, ^5^ In this study we evaluated whether $T_{2}^{*}$ measurements are sufficiently sensitive to detect clinically relevant oxygenation levels in HNSCC at 3T, acknowledging the influence of measurement repeatability, blood volume, and hematocrit.

## Materials and Methods

### Patients and MRI

MR images were acquired in two scanning sessions, between 24 and 168 hours apart, in 10 patients (nine male, one female) with newly diagnosed locally advanced HNSCC, prior to their treatment. A summary of the patient characteristics is presented in Table 1. The median age of the patients was 57 years (range: 44–64 years). Written informed consent was obtained from all patients in this study, which was approved by the Institutional Research Review Board (CCR 3970) and the NHS Research Ethics Committee (REC number 13/LO/0628).

**Table 1:** Summary of Patient Characteristics

Patient no.	Age (years)	Gender	Site	Stage	HPV status	Interval between scans (hours)	Hct	
							MRI1	MRI2
1	57	M	Oropharynx	T3N2bM0	+ve	36	0.300	0.343
2	51	M	Oropharynx	T2N2aM0	+ve	48	0.417	0.411
3	50	M	Hypopharynx	T3N2bM0	−ve	24	0.431	0.418
4	63	M	Oropharynx	T4N0M0	+ve	144	0.459	0.441
5	58	M	Oropharynx	T2N2cM0	+ve	48	0.283	0.354
6	64	M	Supraglottis	T2N2bM0	−ve	144	0.441	0.431
7	64	M	Oropharynx	T2N2c M0	+ve	168	0.437	0.431
8	64	M	Supraglottis	T3N2cM0	−ve	96	0.364	0.400
9	44	M	Oropharynx	T1N2bM0	Unk	168	0.397	0.398
10	51	F	Oropharynx	T3N2cM0	+ve	72	0.389	0.402

*Unk = Unknown human papilloma virus (HPV) status.

MRI was performed at 3T (MAGNETOM Skyra, Siemens Healthcare, Erlangen, Germany) using a dedicated 20‐channel head and neck coil. Patients were aligned in a supine position with slight neck extension using a standard headrest and lateral cushions for improved stabilization. Anatomical coronal *T*_2_‐weighted images (TSE, TE/TR = 76/5000 msec, field of view [FOV] = 250 × 250 mm^2^, 4 mm slice thickness) were obtained first in order to assess the extent of the disease and aid axial sequence planning. Subsequently, *T*_2_‐weighted (TSE, TE/TR = 84/4560 msec, FOV = 240 × 240 mm^2^, 2.5 mm slice thickness) and $T_{2}^{*}$ images were acquired over the volume of interest (VOI) identified by a clinician (L.W. or K.W.). $T_{2}^{*}$ was measured using a 2D gradient echo sequence with six echo times (flip angle [FA] = 24°, TE = 4.92 to 29.52 msec in increments of 4.92 msec, TR = 350 msec, FOV = 240 × 240 mm^2^, 2.5 mm slice thickness, acquisition matrix: 256 × 256, BW = 435 Hz). Echoes were acquired with the same gradient polarity with in‐phase fat and water signal. No signal normalization or filtering was used. All the imaging data were anonymized, coded, and exported to a dedicated research PACS database system (XNAT).^28^ Images acquired from the first MRI session were used as a guide to replicate patient positioning and VOI identification in the second session. A blood sample was taken for a full blood count prior to each MRI scans to determine blood hematocrit.

### MRI Data Analysis and VOI Definition

Paired image datasets were retrieved for image coregistration, VOI definition, and image processing. Signal changes on the multiple gradient echo images were used to calculate 3D $T_{2}^{*}$ relaxivity maps. Data processing was performed using in‐house MatLab software (MathWorks, Natick, MA). Signal intensity decay, measured for increasing echo times, was fitted on a voxel‐by‐voxel basis to a monoexponential model using a least‐squares fit method. No data truncation or filtering was used. Calculated $T_{2}^{*}$ maps were exported in the DICOM format allowing further analysis with a radiotherapy treatment planning system (TPS).

VOIs, including primary and nodal tumor sites, were manually delineated by radiologists (A.M.R. and D.M.K., each having more than 10 years of experience) using the Pinnacle3 TPS (Philips Healthcare, Best, Netherlands). Axial images and $T_{2}^{*}$ maps from both MRI sessions were coregistered using operator‐assisted rigid body algorithms. $T_{2}^{*}$ images with significantly varying neck flexion or translation of tumor volume were identified and the coregistration manually corrected. For such cases primary tumor and involved lymph nodes were coregistered independently to compensate for interscan anatomical and positional variation. $T_{2}^{*}$ values for respective VOIs were exported from the TPS and used for statistical evaluation.

### Statistical Assessment of Repeatability

Tissue $T_{2}^{*}$ values were analyzed using boxplots and $T_{2}^{*}$ difference maps were generated for each VOI to investigate the data distribution and spatial repeatability. Median $T_{2}^{*}$ values were calculated for each VOI and used to assess the quantitative repeatability, to account for a skewed parameter distribution previously described in the literature.^15^, ^19^ First, the Shapiro‐Wilk test was used to ascertain normality of the sample $T_{2}^{*}$ distribution. Second, the Wilcoxon signed rank test was used to compare $T_{2}^{*}$ distributions from each session to check whether the observed interscan variability could be attributed to measurement error. The Bland–Altman method was used to plot the $T_{2}^{*}$ differences between two scan sessions against the mean value of median VOI $T_{2}^{*}$ for both sessions.^29^ Finally, the coefficient of variation (CV) and limits of agreement (LoA, average difference ± 1.96 standard deviation of the difference) were calculated. The potential influence of both the tumor volume and the duration between scans on repeatability was investigated. Kendall's tau (τ) was used to test for correlation between median $T_{2}^{*}$ changes between sessions and VOI volumes, and also between $T_{2}^{*}$ changes and the interval between scans.

#### Simulation of Blood Oxygenation Dependence of $T_{2}^{*}$

A quadratic model was used to describe the blood transverse relaxation rate $R_{2}^{*}$ as a function of fractional blood oxygenation (Y)^8^, ^9^:

(1) $$R_{2}^{*}=A^{*}+B^{*}\left(1-Y\right)+C^{*}{\left(1‐Y\right)}^{2}$$

where A*, B*, and C* are empirically derived coefficients dependent on B_0_ and Hct.

Coefficients A*, B*, C* previously measured empirically for Hct in the range 0.21–0.57 at 3T^9^ were used to obtain $R_{2}^{*}$(Hct,Y) values in the full range of blood oxygenation fractions.

In our simulations, a “tissue hematocrit” (H_tiss_) was used, incorporating the BV fraction and a vascular factor (f_vas_ = 0.85) to account for differences between erythrocyte concentration in large vessels and the tissue capillary network^24^, ^26^:

(2) $$H_{\text{tiss}}=\text{Hct}\times \text{BV}\times f_{\text{vas}}$$

The initial $R_{2}^{*}$(Y) values for Hct in the range 0.21–0.57 were linearly extrapolated to calculate a new set of $R_{2}^{*}$ values at the tissue hematocrit levels. Five blood volume fractions in the range 1–30 ml/100g were considered, including values typical for muscle (BV = 1 ml/100g^30^, HNSCC (BV = 5 ml/100g^31^, and highly vascular tumors (BV > 10 ml/100g^24^. Equation (1) was used to calculate sets of BV‐specific coefficients A*, B*, and C* and to plot the dependence of blood oxygen saturation on $T_{2}^{*}$ values.

A simulated y‐intercept value for BV = 5 mL/100g was subtracted to derive and plot the relative $T_{2}^{*}$ dependence of fractional blood oxygenation and pO_2_ together with corresponding 95% limits of agreement. Relative, rather than absolute, $T_{2}^{*}$ values were used in order to assess the minimum difference in fractional blood oxygenation that can be reliably detected ( $T_{2}^{*}$ changes greater than limits of agreement) and to recognize the effect of tissue‐specific extravascular spin relaxation. Finally, the Hill equation (Hill's coefficient: 2.26, t = 37°C, pH: 7.4) was used to identify the clinically relevant region of hypoxia (pO_2_ < 20 mmHg, Y = 0.32).^6^, ^32^

## RESULTS

### Repeatability

The median time interval between two scans was 84 hours (range: 24–168 hours). For all patients there was no significant difference (*P* = 0.57) in the group mean Hct across the two MRI sessions, with a respective mean value of 0.4 (range: 0.283–0.459). Anatomical image coregistration revealed varying degrees of differences in patient neck flexion between the MRI sessions, despite having attempted to replicate patient positioning at each visit. However, it was possible to compensate for these differences in patient positioning by independent regional coregistration for primary and nodal VOIs.

**Figure 1:**
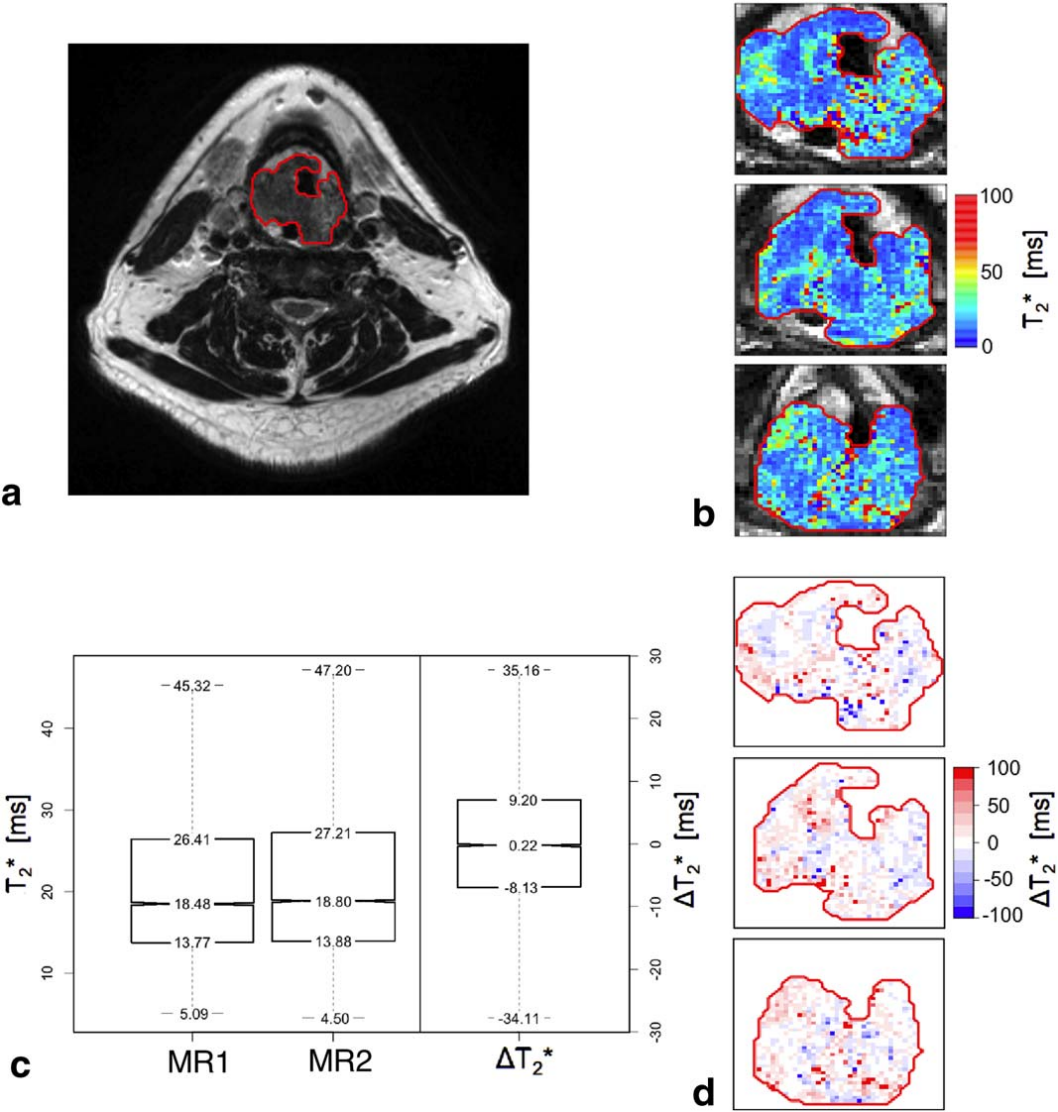
**A**: *T*_2_‐weighted image for a 50‐year‐old stage IV HNSCC patient (patient 3) with outlined primary VOI (red). **B**: $T_{2}^{*}$ parametric maps calculated for visit 1 (three representative slices at the center of the tumor). **C**: Boxplots showing distributions of $T_{2}^{*}$ measured in primary tumor VOI in both visits and differences in $T_{2}^{*}$ between the visits. **D**: Differences in $T_{2}^{*}$ between the visits on a per‐voxel basis.

In total, 22 VOIs were identified and outlined. An example of a VOI overlaid on a *T*_2_‐weighted image is presented in Fig. 1A. One primary tumor and one lymph node (Table 2: patients 1, 10) were excluded from analysis due to inadequate VOI coverage resulting from differences in patient positioning between sessions and/or significant internal motion between *T*_2_‐weighted and $T_{2}^{*}$ examinations. Representative parametric $T_{2}^{*}$ maps, with corresponding differences in $T_{2}^{*}$ between the visits on a per‐voxel basis, and VOI boxplots, are shown in Fig. 1. Table 2 summarizes the measured VOI parameters, including location, volume, $T_{2}^{*}$ values, and differences between the two MR examinations. The mean $T_{2}^{*}$ values significantly (*P* < 0.01) differed between nodes (23.6 msec) and primary tumor sites (18.7 msec). The distribution of intra‐VOI $T_{2}^{*}$ values was positively skewed. An example of the spatial distribution of differences between the relaxation times in two MR examinations (Δ $T_{2}^{*}$) is presented in Fig. 1D. Subregions of uniformly increased or decreased $T_{2}^{*}$ can be observed within analyzed VOIs.

**Table 2:** Summary of Tumor Characteristics

Pt. No.	1° Vol. [cc]	LNs Vol. [cc]	Median T_2_ * [msec]					
			1°			LNs		
			MRI1	MRI2	Δ	MRI1	MRI2	Δ
1	12	15	NA	NA	NA	17.6	20.7	3.1
		2				17.8	21	3.2
2	32	3	18.3	18.7	0.4	25.6	26.7	1.1
3	27	2	18.5	18.8	0.3	24.2	23.3	−0.9
		7				22.9	24.6	1.7
		2				17	18.9	1.9
4	29		21.5	21.5	0.01			
5	173	6	19.2	17.8	−1.4	21.5	20.3	−1.1
		2				21.5	24	2.5
		1				26	26.2	0.2
6	15		16.3	19.7	3.4			
7	6	60	18.6	18.5	−0.2	26.3	26.2	−0.1
8		10				28.4	29.2	0.8
		15				24.6	23.9	−0.8
9		17				21.7	22.5	0.8
10	45	5	16.81	19.19	2.4	NA	NA	NA

1°: primary tumor, LNs: lymph nodes.

*NA = insufficient or poor quality data.

**Figure 2:**
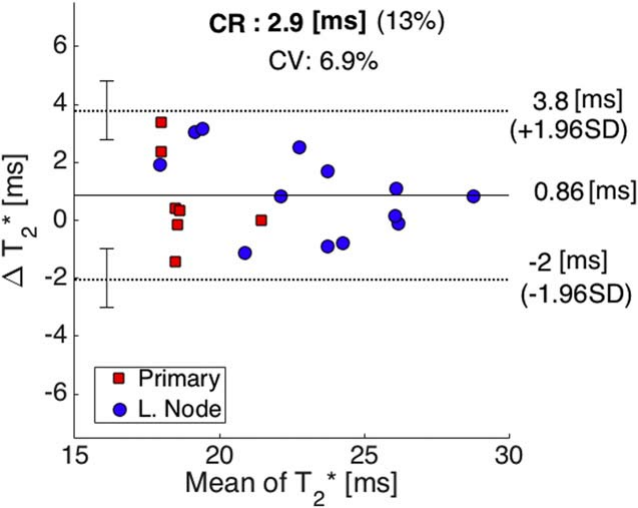
Bland–Altman plot showing difference between median VOI $T_{2}^{*}$ in two scans against the mean value of $T_{2}^{*}$ for both visits (CR = coefficient of repeatability, CV = coefficient of variation). Mean difference (solid line) and 95% limits of agreement (dotted lines) are also shown, with corresponding 95% confidence limits (error bars).

The distribution of $T_{2}^{*}$ for all patient VOIs did not differ from normality (primary tumors: *P* = 0.48, lymph nodes: 0.61) and the population mean values did not differ between the two scan sessions (primary tumors: *P* = 0.32, lymph nodes: *P* = 0.64). A Bland–Altman plot showing $T_{2}^{*}$ difference between the two scans (MR_2_‐MR_1_) against the mean value of VOI median $T_{2}^{*}$ for both sessions is shown in Fig. 2. The coefficient of variation and limits of agreement were 6.9 and 13%, respectively. There was a weak negative and nonsignificant correlation between median $T_{2}^{*}$ differences and the VOI volumes (τ = –0.14, *P* = 0.11), and also between median $T_{2}^{*}$ differences and interval between scans (τ = –0.12, *P* = 0.48).

### Simulations

**Figure 3:**
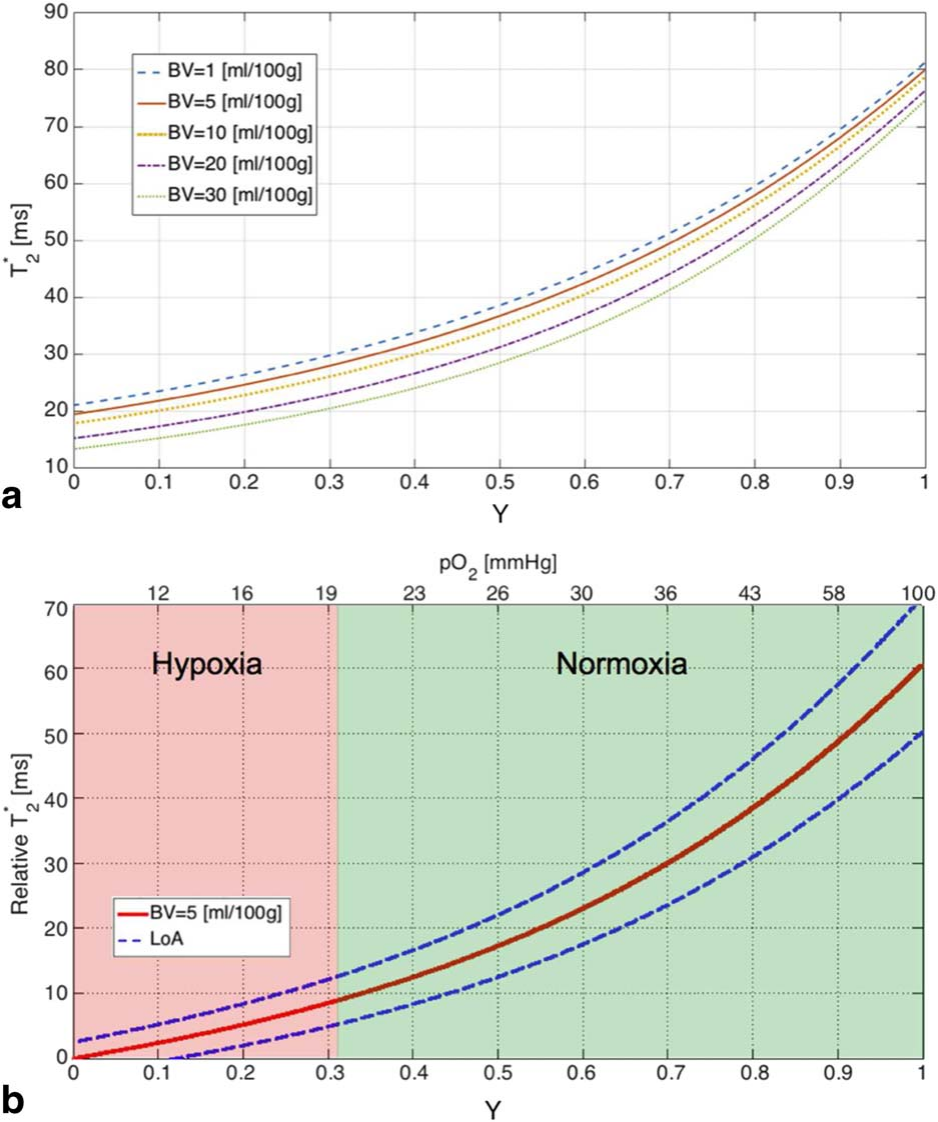
**A**: Transverse relaxation time constant $T_{2}^{*}$ simulated for blood volume fractions in the range 1–30 mL/100g plotted as a function of blood O_2_ saturation (hematocrit: 0.4, microcapillary vascular factor 0.85, field strength: 3T). **B**: Relative $T_{2}^{*}$ dependence of fractional blood oxygenation and pO_2_ simulated for BV = 5 mL/100g. The dashed line shows limits of agreement (LoA, α = 0.05). The oxygen partial pressure was calculated using Hill's equation (human blood, coefficient for blood oxygen binding: 2.26, temperature: 37°C, pH: 7.4).

The average tissue hematocrit levels, H_tiss_, were calculated using a mean of patient Hct values, and for BV = 1, 5 10, 20, and 30 ml/100g were 0.003, 0.017, 0.034, 0.068, and 0.1, respectively. Simulated relative transverse relaxation time constants $T_{2}^{*}$ plotted as functions of fractional blood oxygen saturation in HNSCC are shown in Fig. 3A. The coefficients A*, B*, and C* for BV = 5 ml/100g were 12.42, 19.58, and 17.5, respectively, and the $T_{2}^{*}$_Y=0_ y‐intercept value was 19.4 msec. The relative $T_{2}^{*}$ dependence plot for BV = 5 ml/100g with LoA is illustrated in Fig. 3B. A blood oxygen partial pressure of 20 mmHg corresponded to fractional oxygen saturation of Y = 0.32 and was used as a threshold to identify clinically relevant regions of hypoxia in Fig. 3B. The $T_{2}^{*}$ dependence of fractional oxygen saturation increases monotonically, resulting in increasing sensitivity of the method with increasing blood oxygenation. In normoxic conditions, small changes of ΔY result in $T_{2}^{*}$ changes greater than the repeatability LoA (Δ $T_{2}^{*}$ > LoA). For example, an increase of fractional blood oxygenation from 0.4 to 0.5 would lead to an increase of $T_{2}^{*}$ of 4.8 msec, with the corresponding repeatability threshold of 1.9 msec (LoA = 13%). $T_{2}^{*}$ value differences were lower than the measurement repeatability for fractional blood oxygen saturation below 0.11, or pO_2_ of 12.4 mmHg. For Y values above that threshold, changes in $T_{2}^{*}$ were sufficient to detect differences in blood oxygenation greater than 10% (Δ $T_{2}^{*}$ > LoA for ΔY > 0.1).

## Discussion

MRI measurements of transverse relaxation times have the potential to characterize tissue oxygenation and therefore are of interest in the context of tumor hypoxia imaging. Nevertheless, quantitative tissue oxygenation measurements using BOLD MRI remain a challenge due to $T_{2}^{*}$ dependence on additional biological and physicochemical factors, together with significant intra‐ and intertumor variations. These concomitant independent parameters are likely to explain the moderate correlation between $T_{2}^{*}$, direct measurement of tumor tissue oxygen tension, and immunohistochemical detection of pimonidazole. It is therefore desirable to directly measure these nonoxygen‐related contributions and to stratify tumors into more homogenous subgroups depending on disease site, level of edema, necrosis, and perfusion. Several methods for BV calculation using MRI have been proposed, including measurements of changes in $T_{2}^{*}$ induced by injection of paramagnetic contrast agents such as ultrasmall superparamagnetic iron oxide (USPIO) particles^33^ and gadolinium chelates (dynamic susceptibility contrast [DSC] MRI^34^). To date, USPIO has been investigated clinically as an off‐label intravenous MRI contrast agent with various imaging applications^35^ and could be used in the context of BV measurements. It is also possible to use $T_{2}^{*}$ to study changes in tissue oxygenation as a result of an acute intervention such as blood transfusion, vascular disruptive therapy, or hyperoxic gas challenge, where the significance of relative $T_{2}^{*}$ changes rather than absolute oxygenation levels is of interest.

It is important to recognize that the sensitivity of the quantitative determination of tissue $T_{2}^{*}$ is strongly dependent on measurement repeatability, which in turn is influenced by the iterative shimming process employed. Our data show that a minimum change in median tissue $T_{2}^{*}$ of 13% is required to be regarded as statistically significant within an individual primary tumor or metastatic lymph node. In general, primary HNSCC tumors are expected to be affected by motion and shimming imperfections associated with tissue/air interfaces, and which can explain the increased differences seen in the lower $T_{2}^{*}$ regions. Our model simulation of tissue $T_{2}^{*}$ shows that the sensitivity of the measured change in $T_{2}^{*}$ increases as a function of fractional blood oxygenation. For HNSCC tumors, measured oxygen partial pressure vary widely across a range of 0–70 mmHg, but with a median of between 10 and 20 mmHg.^36^, ^37^ The measured sensitivity of median tumor tissue $T_{2}^{*}$ is thus sufficient to permit detection of clinically significant changes in tumor tissue oxygenation for most HNSCC tumors. However, an exception exists for anoxic, and severely hypoxic, tumor regions (Y < 0.11), where the $T_{2}^{*}$ value differences are lower than the measurement repeatability (LoA = 13%).

Several limitations of our study must be considered. First, BOLD measurements are mainly sensitive to the vascular space and therefore are not suitable for assessment of tissue oxygenation in the absence of functional erythrocyte perfused blood vessels, such as within necrotic tumor cores, which may often be present within metastatic H&N lymph nodes. In addition, there is uncertainty with regard to the appropriate value for the vascular factor (f_vas_) used in the quadratic model of $T_{2}^{*}$ dependence on fractional blood oxygenation.^24^, ^26^ Second, the measured $T_{2}^{*}$ repeatability disregards the presence of true physiological fluctuations in blood flow within the tumor capillary network that lead to transient or cyclical hypoxia, which has been reported in a number of studies.^16^, ^38^ It should be noted that the repeatability of any quantitative MRI biomarker might be influenced by a number of methodological factors, such as patient setup, VOI localization, sequence parameters, and shimming method. In terms of this study, the extent of superior–inferior anatomical coverage used was relatively small (<6 cm), and therefore local shimming is expected to be more repeatable in comparison to large field of view studies in other anatomical sites.^39^ Another limitation of the study was the relatively small number and heterogeneity of tumors imaged (8 primary and 14 metastatic nodal VOIs), with the majority localized in the oropharynx. The distribution of $T_{2}^{*}$ for all patient VOIs, however, was distributed normally, allowing for a reliable repeatability analysis. The accuracy of measured $T_{2}^{*}$ values can also depend on the choice of echo times and type of data processing. In general, the signal‐to‐noise ratio (SNR) in the VOI should be adequate for images acquired using all echo times.^27^ Tissues characterized by short transverse relaxation times suffer from low SNR in the longer echo time images, which may lead to a subsequent overestimation of $T_{2}^{*}$. These effects are not likely to significantly affect the relatively long mean $T_{2}^{*}$ values measured at 3T in HNSCC nodal and primary tumor sites (23.6 and 18.7 msec, respectively). The noise bias should not be neglected when calculating relaxation times of tissues with short $T_{2}^{*}$, or in the case of subregional voxel‐based analysis, in which case Bayesian or data truncation methods might be required.^27^ The number of echoes used herein was a result of using in‐phase fat and water signal with echoes acquired with the same gradient polarity, together with SNR threshold. Previous clinical $T_{2}^{*}$ studies have employed between 4 and 16 echo times.^19^, ^39^, ^40^ In this study, the signal acquired with the gradient echo sequence was found to be dominated by noise for echo times longer than 30 msec. In‐phase fat and water echo times were chosen to account for variable fat content in H&N tumors resulting in potential signal cancellation effects.

Simulations highlight the necessity of additional measurements to enable interpretation of $T_{2}^{*}$ data and quantitative measurements of tissue oxygenation using BOLD imaging, such as blood volume fraction and macroscopic field homogeneity affecting measurement repeatability. Future investigations of $T_{2}^{*}$ as an imaging hypoxia biomarker should include these confounding factors and would benefit from a direct histological verification, which was not available in this study.

The median $T_{2}^{*}$ values measured in the primary tumors were significantly lower than in metastatic lymph nodes, which is likely a consequence of a higher blood volume rather than more severe hypoxia. Similarly, interpretation of lower $T_{2}^{*}$ tumor subregions is possible if the spatial distribution of blood volume is known. The information on tissue oxygenation could be used to assist treatment management, enabling the identification of radioresistant tumors, for which radiotherapy dose escalation, radical surgery, or targeted chemotherapy could be used as an alternative or a supplement of a conventional treatment.

In conclusion, our results confirm the utility of quantitative measurements of $T_{2}^{*}$ at 3T to detect clinically relevant tumor tissue oxygenation across a wide range of BV and oxygen tensions, including those reported for HNSCC. This establishes tumor tissue $T_{2}^{*}$ measurement as a sensitive and reproducible quantitative imaging technique that may be used in future studies of tumor hypoxia.
